# Agricultural Waste-Derived Cellulose/ZnO Composites: Dual Photocatalytic and Adsorptive Action for Textile Dye Removal

**DOI:** 10.3390/polym17131737

**Published:** 2025-06-22

**Authors:** Jihene Belhaj, Ramzi Khiari, Valentín García-Caballero, Antonio A. Romero, Araceli García

**Affiliations:** 1 Nanoval FQM-383 Research Group, Organic Chemistry Department, University of Córdoba, Marie Curie (C-3) Building, Crta. Nnal. km 396, E-14014 Córdoba, Spain; z12bebej@uco.es (J.B.); qo1rorea@uco.es (A.A.R.); 2 National Engineering School of Monastir, University of Monastir, Monastir 5019, Tunisia; 3 Department of Textile, Higher Institute of Technological Studies of Ksar Hellal, Ksar Hellal 5070, Tunisia; khiari_ramzi2000@yahoo.fr; 4 CNRS, Grenoble INP, LGP2, Université Grenoble Alpes, F-38000 Grenoble, France; 5 Departamento de Química Física y Termodinámica Aplicada, Instituto Químico para la Energía y el Medioambiente (IQUEMA), University of Córdoba, E-14014 Córdoba, Spain; g32gacav@uco.es; 6 Instituto Químico Para la Energía y el Medioambiente (IQUEMA), Faculty of Sciences, University of Córdoba, Marie Curie (C-3) Building, Crta. Nnal. km 396, E-14014 Córdoba, Spain

**Keywords:** agriculture waste, photocatalytic degradation, dye adsorption, cellulose/ZnO composites, bromophenol blue

## Abstract

The synthesis of cellulose extracted from agricultural waste, specifically almond and fig tree trimmings, and its combination with ZnO nanoparticles to form cellulose/ZnO composites was studied. These adsorbents/photocatalysts were fully characterized, confirming not only the effective deposition of zinc oxide nanoparticles on the cellulose surface but also the improvement in homogeneity and lower agglomeration and size of ZnO particles grown on these fibers (crystallites were 43 ± 12 nm for pristine ZnO and 13–26 nm for composites). The efficacy of these composites was evaluated against methylene blue (MB), methyl orange (MO), and bromophenol blue (BB), this study being the first time that BB removal results have been reported using dual photo-adsorptive cellulosic composites. After 20 min, removals of approximately 45% were achieved for the anionic dyes MO and BB under UV light and up to 65% for MB with either applied radiation, indicating a clear adsorption mechanism for this cationic dye. A reusability study was conducted for the BB removal system, with only a 15–19% loss in BB removal capacity under UV irradiation after the third reuse. These results demonstrated the potential and efficiency of cellulose/ZnO composites as promising photocatalysts for textile wastewater treatment, providing a sustainable and interesting approach to mitigate dye pollution.

## 1. Introduction

The global textile industry plays a key role in the global economy. As of 2024, its market size is estimated at approximately USD 1.7 trillion, with projections suggesting growth to more than USD 3 trillion by 2030. However, the fashion sector remains the second highest industrial contributor to waste generation and pollution, accounting for 8% of global carbon emissions and 20% of all wastewater [1]. The textile industry consumes approximately 93 billion cubic meters of water, equivalent to the annual consumption of 5 million people. This large water usage is primarily caused by dyeing and finishing processes, which require substantial amounts of water for fabric treatment and coloration. Over the past two decades, significant efforts have been made to address the environmental impact of dyes in industrial wastewater [1,2,3] due to the large volumes generated and the vast array of compounds involved. More than 100,000 types of dyes are commercially available, with annual production exceeding 700,000 tons. Due to their high solubility, synthetic dyes are common water pollutants, often present in trace amounts in industrial wastewater [4]. As organic content regulations become more stringent, the removal of dyes before discharge is crucial. Many of these dyes are toxic and pose serious risks to water quality, plant and animal life, and human health, for which prolonged exposure has been linked to skin and eye irritation, corneal and conjunctival lesions, dermatitis, and even cancer [5].

In recent years, there has been a strong research trend in the development of new biomass-derived materials as photocatalysts for the treatment of this textile-contaminated industrial water [6,7,8]. One of these promising materials is cellulose, a biodegradable and renewable polymer derived from agricultural and food waste that has been extensively studied for its potential in various applications, including water purification [9] or energy storage [10]. As an improvement, researchers have explored the synthesis of cellulose-based composites with other materials, such as metal oxides [11,12,13,14] in a symbiosis that combines the best of a biodegradable and sustainably sourced matrix with the proven dye-removing efficacy [4,15,16] and the antibacterial activities of metal oxides [17,18,19], with even more effective properties achieved by combining the two through the development of cellulose and metal oxide composites [20].

ZnO, together with TiO_2_ particles, is one of the semiconductors most extensively studied as photocatalysts [21,22], gaining considerable attention for its low production cost, biocompatibility, narrow bandgap energy, flame retardancy capacity, and other beneficial properties [23,24]. In addition to its catalytic properties, ZnO has been used in the adsorption of organic dyes [25] because due to the ionization of Zn−OH groups on its surface, this material has a point of zero charge (PZC) between 9 and 11 and favors electrostatic attraction and hydrogen bond attraction, π–π interactions, and Van der Waals forces [24], improving the adsorption of anionic molecules (such as methyl orange, bromophenol blue, or amaranth) in acidic media or cationic molecules (methylene blue) under basic conditions. Although ZnO is capable of adsorbing dyes, the better the greater the porosity and specific surface of the material, its greatest advantage is that it acts as a photocatalyst, degrading dyes under UV or visible irradiation. In these processes, the dye is first adsorbed and then degraded by reactive species generated by ZnO.

However, the efficiency and applicability of ZnO is limited by its agglomeration tendency [26], which significantly affects its photocatalytic performance [23,27]. One solution involves the deposition of ZnO particles on a polymeric support or substrate [28], which provides an active surface that allows for a homogeneous dispersion of the nucleation sites and prevents particle accumulation [23,29] in addition to improving its photocatalytic properties, such as a broadened absorption edge and a wider wavelength range to achieve pollutant removal. This solution, using different types of cellulose (different size, functionality, plant or bacterial origin…) has become of great interest in last few years [21,30], with researchers seeking a direct application in the removal of coloring molecules used in the textile industry, such as crystal violet, methylene blue, methyl orange, or other aqueous contaminants such as phenols or pharmaceuticals.

To the best of our knowledge, there are no published works evaluating the removal of these three different textile dyes using cellulose composites with zinc oxide. The present study focuses on the synthesis of cellulose/ZnO composites derived from agri-food wastes for the removal of textile dyes by an alkaline deposition method using zinc acetate dihydrate as a precursor. The different materials were analyzed to determine their structure, morphology, and chemical functionality (surface charge, FT-IR, XRD, XPS, and porosimetry). Furthermore, the optical properties (bandgap) of the obtained cellulose/ZnO composites were evaluated and compared with those of ZnO particles prepared by the same method. Finally, their ability to remove dyes was evaluated, studying the two possible mechanisms that can occur and their potential synergy: adsorption (tests conducted in the dark) and photodegradation (experiments irradiated with UV and white light) as well as how the properties of the materials evaluated influence them. The use of agricultural and food waste-derived cellulose as a precursor for the synthesis of these composites offers a sustainable and environmentally friendly approach to the development of less sustainable or more harmful materials.

## 2. Materials and Methods

### 2.1. Materials

Two agricultural residues were used as cellulose source, namely almond (*Prunus amygdalus* L.) and fig (*Ficus carica* L.) trimmings. The raw materials were collected in January 2022 in Ksour Essaf, Mahdia, Tunisia. After harvesting, the agricultural wastes were dried under ambient conditions (65% humidity, 25 °C) until constant weight, after which sand was removed by conventional washing and finally dried again under the previous conditions prior to their use.

For the preparation of cellulose and cellulosic composites, sodium hydroxide pellets (≥99.0%, AGR, from Labkem), hydrogen peroxide (30%, GLR, from Labkem), and zinc acetate dihydrate (≥98%, ACS, from Labkem) were used as supplied. Deionized water was used for washing and sample preparation.

The performance of cellulosic composites in water decontamination processes was evaluated using methyl orange (MO, C.I. 13025 ACS), methylene blue (MB, C.I. 52015 IND), and bromophenol blue (BB, IND ACS), all supplied by Labkem (Barcelona, Spain) and used as received.

### 2.2. Cellulose Extraction

The raw materials were first chipped into uniform pieces in an automatic jaw crusher with 0.5 cm sieve (Retsch GmbH, Haan, Germany). Then, a pressurized and thermostatized 15 L reactor (Metrotec Techlab Systems, Lezo, Spain) was used to delignify the chips with an 8:1 liquid/solid ratio and 10% NaOH at 170 °C for 60 min. The treated material was subsequently thoroughly washed, disintegrated in a PFI grinder (Metrotec Techlab Systems, Lezo, Spain) at 1200 rpm for 30 min, filtered through a 50 µm mesh, and oven dried (105 ± 3 °C) until constant weight [31]. The resulting pulp was bleached to remove impurities such as proteins, phenolic compounds, and remaining lignin. The bleaching process started with 30 g of dry fiber mixed with 200 mL of 5% H_2_O_2_ aqueous solution and 2–3 mL of 15% NaOH to raise the pH and was conducted for 120 min at 80–85 °C, followed by washing and filtration. This step was repeated 2–3 times until complete bleaching. The bleached fibers obtained from almond tree and fig tree trimmings, hereinafter abbreviated as ATcell and FTcell, respectively, were then oven dried at 105 ± 3 °C until constant weight and hermetically stored for later use.

The morphology of ATcell and FTcell fibers was assessed using a MORFI (LB-01) analyzer, developed by Techpap (Gières, France). This analysis equipment uses an image analysis technique to determine the main morphological parameters of a fiber suspension in terms of the arithmetic mean of fiber length (L, mm) and fiber width (W, μm). The measurements were conducted in triplicate with 1 L of aqueous of 0.03% wt. of cellulosic pulp [32].

### 2.3. Cellulose/ZnO Composite Preparation

Cellulose/ZnO composites were prepared by an in situ solution casting technique, using 2.2 g of previously prepared cellulose fibers dispersed in 100 mL of 0.5 mol·L^−1^ zinc acetate dihydrate aqueous solution. The mixture was heated at 90 °C for 60 min under continuous magnetic stirring [19]. Subsequently, 0.5 mol·L^−1^ NaOH was dropwise added to the solution while maintaining the same conditions (90 °C and the magnetic stirring) until a precipitate was formed, then recovered by vacuum filtration (nylon membrane filters of 0.45 μm, Ø 47 mm, Labbox, Barcelona, Spain) and thoroughly washed with deionized water and oven dried (100 ± 3 °C for 24 h), ensuring the complete formation of ZnO without intermediate compounds [33]. This procedure was applied for ATcell and FTcell samples, obtaining the corresponding ATcell/ZnO and FTcell/ZnO cellulosic composites. The inorganic matter (IM) of the prepared celluloses and cellulosic composites, which should be considered entirely due to the zinc oxide deposited on cellulose surface, was determined by gravimetry after combustion of each sample in a ceramic crucible at 575 °C for 4 h in a laboratory furnace [34]. The IM was calculated according to Equation (1).

(1) $$IM\left(\%\right)=\frac{m_{f}-m_{i}}{m_{s}}\times 100$$

where m_f_ and m_i_ are the mass (g) of dry crucible with sample after and before burning and m_s_ is the mass of sample. This measurement was performed in duplicate for each sample.

### 2.4. Sample Characterization

For detailed information about performed analytical techniques, please refer to Supplementary Information (SI) Text S1 [35,36].

### 2.5. Removal of Dyes

Experiments were conducted in triplicate with 0.1 g of each cellulosic sample suspended into 50 mL of 5 ppm aqueous solution of each dye, i.e., methylene blue (MB), bromophenol blue (BB), and methyl orange (MO) [37]. Each experiment was kept for 20 min under constant stirring (200 rpm) in a photoreactor prototype [38], and different light sources/conditions were considered: UV light, white light, and darkness (see Text S2 in SI for more details). Removal of dyes was monitored by collecting aliquots at regular intervals and analyzed using a UV/VIS spectrophotometer Lambda 365 UV-Vis spectrophotometer (PerkinElmer, Waltham, MA, USA) after filtration with syringe nylon filters (0.45 µm).

The absorbance spectra of the supernatants were recorded at wavelengths of 664 nm, 598 nm, and 464 nm for MB, BB, and MO removal experiments, respectively, corresponding to the respective maximum wavelength λ_max_ (see Table S1). Dye removal (R%) was calculated based on the recorded absorbance according to the Equation (2).

(2) $$R\left(\%\right)=\frac{\left({ABS}_{0}-{ABS}_{t}\right)}{{ABS}_{0}}\times 100$$

where ABS_0_ and ABS_t_ are the absorbances of the supernatants at the beginning and at each time of the experiment, respectively.

### 2.6. Regeneration and Reuse of Composites

From a sustainability perspective in the treatment of wastewater from the textile industry, the regeneration and recycling of adsorbent and catalytic materials results in more than necessary [29]. After each performed test, the composite was recovered by vacuum filtration (low ash content filter paper, BRANCHIA by PRAT-DUMAS equivalent to Whatman 52), rinsed 4 times with deionized water to remove residual non-adsorbed dye [39], and dried (24 h at 80 °C) for its analysis and subsequent regeneration/reuse study.

The reusability of cellulose/ZnO composites was evaluated for BB removal system by treating the adsorbed catalyst with 5 mL of absolute ethanol [29] followed by centrifugation (3000 rpm, 10 min) and ethanolic solution removal. This was carried out 3 times, after which the material was dried in an oven (24 h, 80 °C) before being reused. The reusability of the composites was conducted in duplicate.

### 2.7. Statistical Analysis

The dye removal process was carried out in duplicate. The results were expressed as mean values ± standard deviation. The evaluation on the influence of different parameters over dye removal assays was assessed by multiple variable analysis using the software STATGRAPHICS centurion XV, version 15.2.11 (StatPoint, Inc., Warrenton, VA, USA). Pearson correlation parameters and *p*-values were calculated for each pair of variables, with *p* < 0.05 reflecting significance, in order to assess the linear and significant relationship between them.

## 3. Results and Discussion

### 3.1. Characterization of Samples

Cellulose fibers from pruning wastes of almond and fig trees were obtained in a previous work [32]. As shown in Table 1, the morphology of ATcell and FTcell fibers was very similar to each other (0.54–0.57 mm in length and 19.3–22.7 μm in width) and coincided with most fibers obtained from annual plants in conventional biomass fractionation biorefinery processes. Several studies have been reported in the literature indicating that the size of cellulose fibers may be a determining factor in the deposition of ZnO particles on their surface. Li et al. [26] found that morphology affects the chemical deposition of ZnO, yielding higher content with the decrease in fiber size. Similarly, other authors concluded that not only the amount but also ZnO particle size and morphology [5] are directly related and can be controlled by varying the diameter of micro/nano-cellulose fibers with a grinding treatment [40]. Considering the present results on fiber size, no major difference in the ZnO content of the composites prepared from ATcell and FTcell would be expected since both materials have similar sizes.

This behavior is clearly demonstrated by the inorganic (IM in Table 1). Both ATcell and FTcell presented ash contents below 2% wt., attributed to residues of chemicals used during their production. After alkaline treatment with zinc acetate, the samples presented inorganic contents of approximately 47% in both ATcell/ZnO and FTcell/ZnO, which indicated that the growth performance of zinc oxide particles on their surface was almost equal.

The presence of surface functionalities, especially negatively charged ones such as hydroxyl groups, can serve as a zinc oxide nanoparticle growth point and improve their dispersion on the surface of a substrate [15]. Thus, as asserted by Li et al. [40], the uniform nucleation Zn^2+^ to form ZnO particles on cellulosic fibers strongly depends on the preparation pathway and the existence of −OH groups on the cellulose surface. As confirmed by the conductometric titration results in Table 1, ATcell and FTcell celluloses showed certain surface charges of 51.0 and 79.81 μmol/g, respectively, due to hydroxyl and acidic groups, the last probably formed during the samples’ preparation [41], confirming the great importance of the origin of the biomass in the behavior of the cellulose extracted from it. For the cellulose/ZnO samples, this charge increased considerably to 340–400 μmol/g, proving again the chemical deposition of ZnO particles (836.2 μmol/g) on the surface of the fibers since these are strongly positively charged [25].

**Figure 1:**
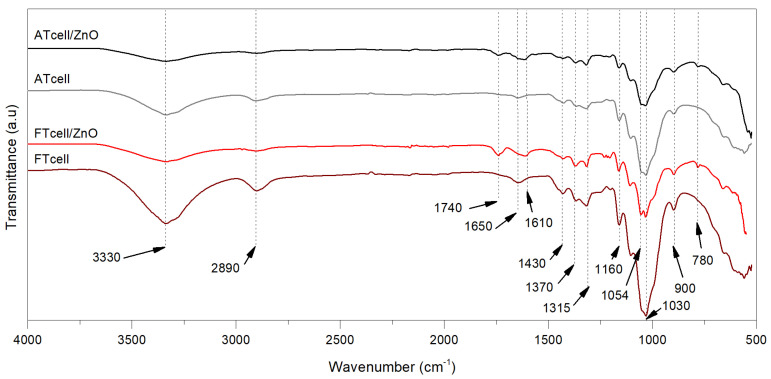
FT-IR for obtained celluloses (ATcell and FTcell) and prepared composites (ATcell/ZnO and FTcell/ZnO).

The FT-IR spectra of cellulosic samples are shown in Figure 1. The peaks at 3330 cm^−1^ and 2890 cm^−1^ represent the stretching vibration of hydroxyl and methyl groups in polysaccharides, such as cellulose. Additional absorption bands at 1430, 1370, 1315, 1160, and 1030 cm^−1^ are associated with stretching and bending vibrations of −CH_2_, −CH, −OH, C−O, and C−O−C bonds in cellulose [39]. The 1430 cm^−1^ band indicates the amount of crystalline structure (less intense after functionalization with ZnO), while the 900 cm^−1^ band represents β-glycosidic linkages between glucose units in cellulose [41].

Although ZnO vibration modes have been reported to appear in the range of 500–400 cm^−1^ [42], some IR bands detected in the present study have already been related to the formation of ZnO particles and their interaction with the cellulose surface [5]. Two bands at 1050 cm^−1^ and 780 cm^−1^ have been assigned in the literature to successful ZnO formation and its interaction with cellulose [13,16], appearing in both cell/ZnO spectra but being more noticeable for FTcell/ZnO. The interaction of ZnO particles with the cellulose structure can also be considered by the intensity reduction in the broad −OH band at 3300 cm^−1^, suggesting that ZnO particles not only have a strong interaction with the −OH groups of cellulose [5,13,22], but also that the intensity reduction would be due to the growth of the oxide particles directly on the hydroxyl groups available on the cellulose surface. This has been previously proposed in the literature [25,26], thus resulting in the reduction of the band at 3300 cm^−1^ being more evidence of the successful deposition of ZnO on ATcell and FTcell fibers. The significant change on the 2890 cm^−1^ band of cellulose/ZnO composites has been related to the successful functionalization of cellulose fibers with ZnO [5], probably due to hydrogen bond interactions or the formation of cellulose acetate. This last mechanism could be justified by the appearance of a band at 1740 cm^−1^ in both ZnO composites, which has been related in the literature to the stretching vibration of the carbonyl in acetyl groups [14]. In the present work, a slight shift of the band from 1650 cm^−1^ to a lower wavelength in ATcell and FTcell samples was observed in the cellulose/ZnO composite samples, related to the presence of water in the cellulose structure.

Optical microscopy was used to examine the morphology and distribution of pure ZnO particles and cellulose/ZnO composite materials at three different magnifications. As observed in Figure S2 in SI, the ZnO particles had irregular, almost spherical shapes, with a marked tendency to agglomerate due to their high surface energy and Van der Waals interactions. Particle sizes varied from submicrons to a few microns. However, whatever the fiber used, the cellulose/ZnO composites showed a more homogeneous distribution of ZnO particles within the cellulose fiber. The ZnO is largely present on the surface of the fiber. These observations suggest good interfacial compatibility between the two components and good incorporation of the ZnO into the cellulose structure.

**Figure 2:**
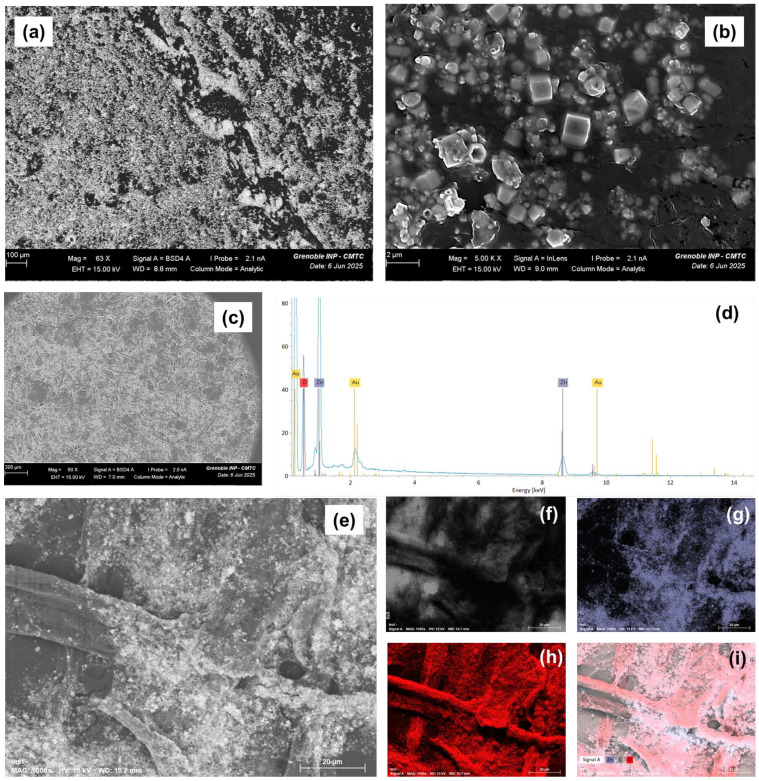
SEM imaging of ZnO (**a**,**b**) and FTcell/ZnO samples (**c**); elemental analysis spectrum of ZnO sample (**d**); EDX images (**e**); and mapping of ATcell/ZnO composite: C (**f**), Zn (**g**), O (**h**), and whole (**i**) (carbon in grey, zinc in blue and oxygen in red).

The morphological and microstructural changes in the cellulose/ZnO composite modified with ZnO particles were also examined using scanning electron microscopy (SEM), and the results are shown in Figure 2. The images reveal significant alterations in the external surface of the cellulose fibers. Following the saturation of the fibers with ZnO precursors and the in situ synthesis of nanoparticles, a new surface layer is formed. This layer exhibits increased roughness and noticeable nanoparticle agglomeration [5].

To assess the uniformity of the ZnO film deposition, SEM imaging was used to evaluate the surface morphology and particle distribution across the composite. The homogeneity of the ZnO nanoparticle layer was determined based on the consistency of the coating and the absence of large uncoated areas or excessive clustering. Additionally, energy-dispersive X-ray spectroscopy (EDX), coupled with SEM, was employed to confirm the elemental composition and spatial distribution (mapping) of zinc (Zn) and oxygen (O) across the fiber surface. Figure 2 further supports these observations by presenting the ATcell/ZnO composite that exhibits a uniform distribution of ZnO nanoparticles and successful grafting onto the cellulose fibers (Figure 2e–i).

**Figure 3:**
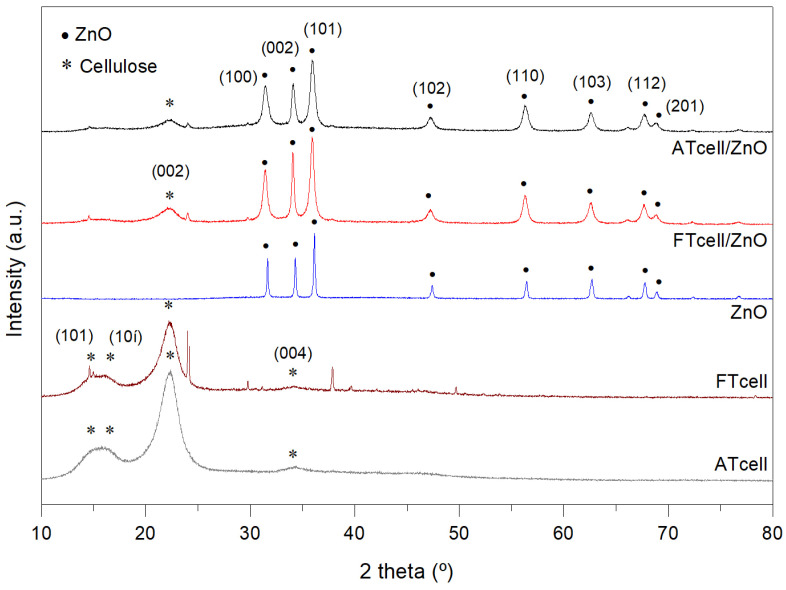
XRD patterns for ATcell, FTcell, ATcell/ZnO, ATcell/ZnO, and ZnO samples (peaks identified with * and with • for cellulose and ZnO, respectively).

The structural evaluation on the prepared samples was obtained by XRD analyses. The diffractograms for cellulose, ZnO, and cellulose/ZnO composites are represented in Figure 3. ATcell and FTcell samples exhibited characteristic diffraction peaks of cellulose Iα (JCPDS Card No. 56-1719) with a shoulder containing overlapped signals at 14.7° and 16.2° (for (101) and (10ī) planes), a strong peak at 22.6° (002), and a weak signal at 34.3° (004) [18,26]. Cellulose extracted from almond trimmings (ATcell) and fig trimmings (FTcell) presented similar crystallinity values of 66.32% and 66.42%, respectively (Table 1). On the other hand, XRD patterns for the prepared ZnO sample displayed peaks at 31.68°, 34.32°, 36.15°, 47.41°, 56.46°, 62.71°, 67.79°, and 68.93° assigned to (100), (002), (101), (102), (110), (103), (112), and (201) characteristic planes of the hexagonal wurtzite structure of ZnO, respectively (JCPDS Card No. 36-1451). These peaks also presented in the diffractograms of ATcell/ZnO and FTcell/ZnO samples, proving that ZnO particles were successfully incorporated onto the cellulosic surface.

Changes in the intensity and width of the diffraction peaks of ZnO grown on the cellulosic surface can be observed, which according to Zhang et al. [30] demonstrates that this substrate provides a large number of potential nucleation sites, reducing the stacking or agglomeration of ZnO particles. On the other hand, a significant decrease in cellulose-related peaks and crystallinity was observed for ATcell/ZnO and FTcell/ZnO composites (49.68% and 52.21%, respectively), being attributed to the presence of ZnO particles covering the cellulose surface and hampering the measurement [5,26]. According to Onyszko et al. [5], it was confirmed that the formation of zinc oxide was complete and without any traces of Zn(OH)_2_ as intermediate product, since as they stated it presents some characteristic XRD peaks in the range of 10–30°, which were not found for our composites.

According to the Debye–Scherrer equation, the average size of ZnO crystallites in ZnO, ATcell/ZnO, and FTcell/ZnO samples was 43 ± 12 nm, 16 ± 3 nm, and 19 ± 7 nm, respectively (see Table S2). These results were in agreement with those of Li et al. [40], who reported similar particle sizes for ZnO powder (40.61 nm) and lower values for cellulose/ZnO composites (14–16 nm). In the literature, authors argued that the growth of ZnO on the cellulosic substrate allows for a better dispersion of oxide particles, which avoids agglomeration into a larger particle size and further a decrease in ZnO crystallites on the composite surface, improving photocatalytic performance [26,27,40]. In addition, the lattice strain ε was also calculated to evaluate possible distortion or deformation of the formed ZnO crystals. Thus, for the ZnO sample, an ε average value of 2.1 ± 0.2×10^−3^ was found (Table S2), whereas higher lattice strains of 5.6 ± 1.6×10^−3^ and 4.9 ± 1.2×10^−3^ were calculated for ATcell/ZnO and FTcell/ZnO samples, respectively. This strain increase is not considered severe and might be due to a reduction in particle size, a fact confirmed by the crystallite size values discussed above, but also to crystalline defects due to vacancies in the lattice or dislocations due to an interaction between the ZnO and the cellulose substrate that might produce these crystal deformations [28].

**Figure 4:**
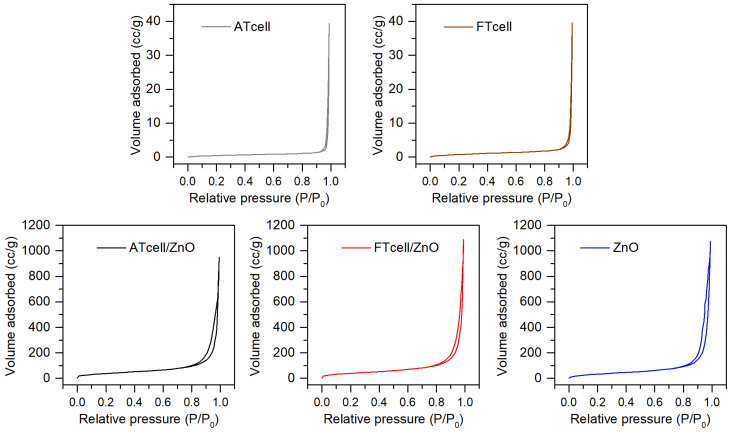
N_2_ adsorption/desorption BET isotherms for the prepared samples.

Adsorption/desorption isotherms (see Figure 4) and textural properties such as BET surface area and BJH average pore size (Table 1) of cellulose and cellulose/ZnO samples confirmed the success of ZnO particle formation onto cellulose surface. The original ATcell and FTcell samples were found to be macroporous materials, with low surface area of 1.8 and 3.3 m^2^/g and few large pores (79.8 and 56.3 nm, respectively, with BJH cumulative pore volume of 0.05 cc/g). On the other hand, ATcell/ZnO, FTcell/ZnO, and ZnO samples presented a mesoporous behavior with 14.5, 14.9, and 13.4 m^2^/g surface area and pores of 33.2, 36.1, and 38.1 nm in diameter, respectively.

Thus, the incorporation of ZnO allowed for an increase in the porosity of celluloses (up to 0.16 cc/g BJH cumulative pore volume), increasing the BET surface area, with more but smaller pores. However, the here-prepared ZnO material results were less porous than those obtained by Zafar et al. [25], even following a similar synthesis pathway, a condition that is considered key in the morphology and structure of ZnO particles, as it severely affects the nucleation and crystal growth, essential for controlling the content of the ZnO in the final composite and its photocatalytic behavior [18].

**Figure 5:**
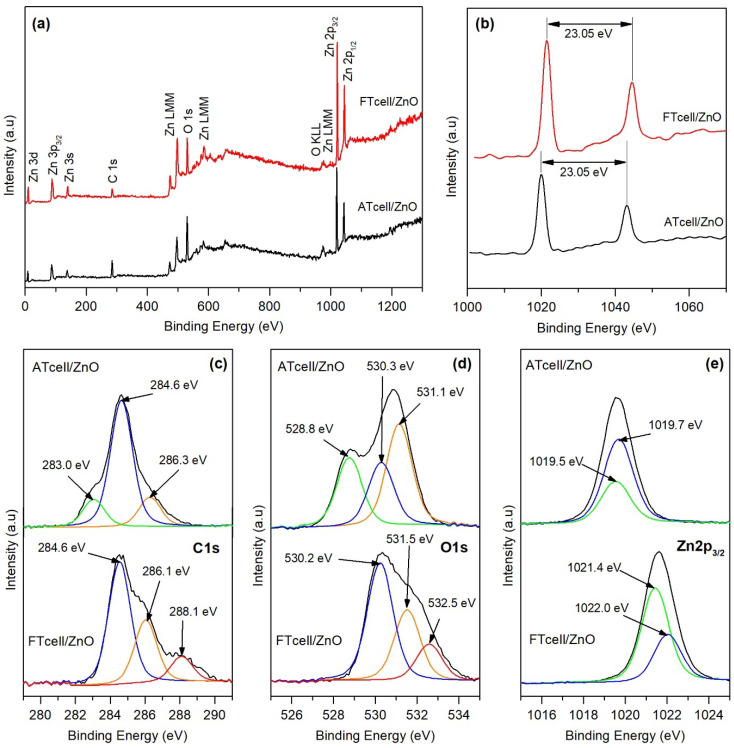
XPS survey scan full spectra of cellulose/ZnO composites (**a**), detailed spectra of Zn 2p signal (**b**), and high-resolution XPS spectra for (**c**) C 1s, (**d**) O 1s, and (**e**) Zn 2p3/2.

XPS survey spectra confirmed the presence of carbon (C 1s), oxygen (O 1s), and zinc in both ATcell/ZnO and FTcell/ZnO composites, as depicted in Figure 5a. For zinc, Zn 3d, Zn 3p, Zn 3s, several Zn LMN, and Zn 2p peaks were identified. Auger peaks occur when an excited atom releases energy without emitting a photon, which depends only on the electronic structure of the element and not on its chemical environment as in XPS peaks; thus, they help to distinguish between different oxidation states. In ZnO samples, an Auger peak located at 999 eV corresponds to samples containing ZnO (Zn^2^⁺). In the spectrum, a peak close to 976 eV was also observed, which, although it could be attributed to a metallic Zn^0^ state, is also characteristic of the KLL peak of oxygen in the O^2−^ state in metal oxide samples.

The signal doublet at ~1021 and ~1044 eV (Figure 5b), identified as the Zn 2p3/2 and Zn 2p1/2 lines, respectively, showed a characteristic spin-orbit separation of 23.05 eV, indicating a normal state of Zn^2+^ in ZnO [17,18]. The shift towards higher binding energies of the Zn 2p peaks has been observed before in the literature, being related to higher oxidation states of zinc [33] in the form of Zn(OH)_2_ (which would indicate an incomplete formation of ZnO during the preparation of the composites) but also to a strong interaction of the ZnO particles with the hydroxyl groups of cellulose [27,43]. Nevertheless, in the present work, a shift towards lower binding energies was observed for the ATcell/ZnO (1019.8 and 1043.3 eV), which could indicate the coexistence of Zn^0^ on the surface of the sample. The formation of metallic zinc is unlikely since no work has been reported where this occurs on any substrate without a calcination step during the preparation of nanoparticles [44], although the possible occurrence of changes in the normal error margin during the analysis has also been discussed [43].

The high-resolution XPS of C 1s for cellulose/ZnO composites demonstrated that several chemical states of C were present (Figure 5c), yielding characteristic binding-energy peaks at 284.6 eV and at around 286 eV assigned to C–C/C–H and C–O in the alcoholic/ether groups in the cellulose structure, respectively [26]. The lower binding 283.0 eV peak that appears for the ATcell/ZnO sample has been reported as the presence of carbon atoms in carbide form, indicating the formation of Zn–C bonds [44]. Thus, the formation of C–Zn interactions becomes more plausible than the possible formation of metallic zinc (lower energy shift observed above for the Zn 2p peak) on the cellulose surface. Cho et al. [44] corroborated the replacement of oxygen by carbon in the ZnO crystal structure by observing the lattice expansion in their XRD results. In the present work, higher values for the lattice strain than those previously reported for pure ZnO were found for both cellulose/ZnO composites (Table S2), being especially higher for ATcell/ZnO in the 002 plane, corresponding to the c-axis growth perpendicular to the substrate. On the other hand, for the FTcell/ZnO composite, a higher binding energy peak was recorded at 288.1 eV that could indicate the existence of C=O bonds in the surface of the sample, probably related to carboxyl groups [26,30], which agrees with the higher surface charge obtained above for the pristine FTcell sample.

The XPS spectrum of O 1s (Figure 5d) shows a lower binding peak at 528.8 eV for the ATcell/ZnO sample that indicates a strong interaction between oxygen and zinc forming the metal oxide. It also displayed a peak near 530 eV for both samples that has been reported as the characteristic O^2−^ ion in the hexagonal wurtzite structure of ZnO [29,31], thus confirming the formation of ZnO on both cellulosic samples. The peaks at around 531.1 and 531.5 eV for ATcell/ZnO and FTcell/ZnO, respectively, could be attributed to defects on the ZnO structure due to chemisorbed oxygen [27] and oxygen vacancies but also to the presence of C–O–Zn bonds [30], which corroborates the anchoring and growth of the ZnO particles in the hydroxyl groups of the cellulose surface [17]. The highest binding energy in the FTcell/ZnO spectrum (532.5 eV) is in fact due to the free C–OH groups on the substrate surface [24], indicating a more efficient nucleation of ZnO in the ATcell/ZnO sample, which presented lower ρ values and therefore a lower amount of hydroxyl groups on the surface. The measured atom % moieties (at ~10 nm dept on sample surface) by XPS analysis for carbon, oxygen, and zinc at their main peaks (located at around 286 eV for C 1s, at 530 eV for O 1s and at 1021 eV for Zn 2p_3/2_, respectively) resulted in 16.0% C, 22.5% O, and 61.5% Zn for ATcell/ZnO and 26.4% C, 26.4% O, and 47.2% Zn for FTcell/ZnO. All this confirmed that there was a clear difference in the composition and functionality between the original ATcell and FTcell samples, resulting in significant differences in the surface of the zinc oxide composites.

**Figure 6:**
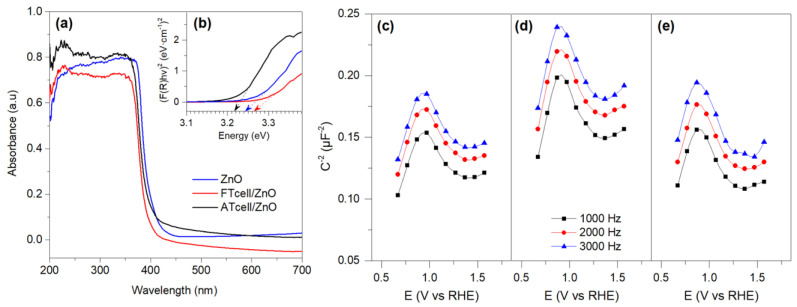
UV–vis DRS spectra of ZnO and cellulose/ZnO composites (**a**), Tauc plots of bandgap energy calculation (**b**), and Mott–Schottky plots of ZnO (**c**), ATcell/ZnO (**d**), and FTcell/ZnO (**e**) samples.

To evaluate the photocatalytic activity of a material, it is essential to know the range of the electromagnetic spectrum it can absorb and the minimum energy required to excite electrons from the valence band to the conduction band, i.e., the bandgap. The optical response of the prepared materials is shown in Figure 6. 

By comparing the prepared ZnO particles and cellulose/ZnO composites, the light absorption intensity of the cellulose/ZnO composites increased, and it was found that the cellulose/ZnO composites revealed excellent UV absorption properties in the visible region. As shown in Figure 6a, the absorption edge of the cellulose/ZnO composites was found to shift below 400 nm, indicating a decrease in the bandgap energy of the pure ZnO.

As illustrated in Figure 6b, bandgap energies (E_g_, eV) of the ZnO, ATcell/ZnO, and FTcell/ZnO composites were estimated to be 3.24, 3.22, and 3.27 eV, respectively. Thus, all the analyzed materials presented a bandgap close to 3.2 eV, indicating that they are suitable to work in the UV light range so that the photogenerated electron–hole pair would react with OH groups (from water and/or the surface of the materials) to achieve the degradation of contaminants in an aqueous medium.

The bandgap value obtained for the prepared ZnO particles is in agreement with the results reported in the literature [21,23,30,44], although this energy depends greatly on the size of the metal oxide particles. Li et al. [26] also observed a small decrease in the bandgap energy for cellulosic composites with zinc oxide compared to pristine ZnO particles, which they attributed to the surface charge of the initial fiber that improves the deposition of zinc oxide. Likewise, as Cho et al. suggest in their work [44], certain interactions with doping materials in vacancies of the ZnO crystal structure would produce a reduction in the absorption energy and bandgap of the material. Thus, crystallites with vacancies and defects in their structure could enhance the interaction of ZnO with light radiation (hv), improving its photocatalytic activity since oxygen vacancies generate energy levels within the ZnO bandgap, facilitating the absorption of lower-energy photons. This can broaden the spectral response of ZnO particles into the visible region, improving their activity under sunlight. Here, the greater presence of O^2−^ vacancies in the crystalline structure of the ZnO particles, previously observed in the XPS results, together with a lower agglomeration of the same according to the XRD measurements carried out, could explain the reduction of the bandgap for the ATcell/ZnO sample. In this way, cellulose composites demonstrated good visible light harvesting capabilities compared to ZnO particles, probably due to the charged and OH-rich surface of cellulose acting as a sensitizer in bandgap modulation on composites.

According to the Mott–Schottky analysis and as can be seen in Figure 6c,d, all the materials used in this work present the typical behavior of an n-type semiconductor. In this way, we can approximate the conduction band limit of the different semiconductors. The flat band (where C-2 becomes 0 V) of ZnO is more negative (0.1 V vs. RHE) than those of the ATcell/ZnO and FTcell/ZnO samples (0.2 V vs. RHE), which is a shift to positive potentials of 0.1 V. This now allows us to approximate the conduction band of ZnO to −3.77 eV and that of the ZnO-modified celluloses to −3.87 eV [45]. Due to the fact that this difference in potential in the conductivity band of the different samples is very small, it cannot be related to a higher degradation of the different dyes studied in this work, which could mean that the higher degradation of the dyes is due to a smaller particle size in the ZnO-modified cellulose samples than the pristine ZnO itself.

### 3.2. Dye Removal Performance

**Figure 7:**
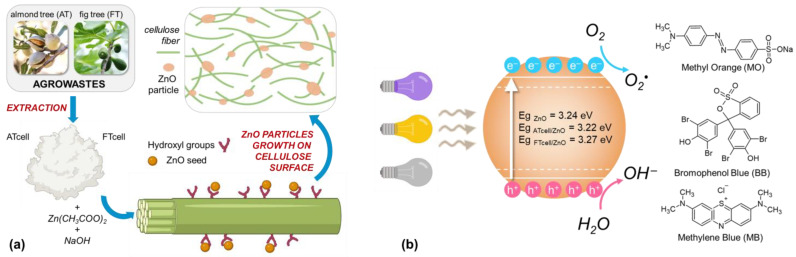
The proposed mechanisms for cellulose/ZnO composite synthesis (**a**) and for the photodegradation of the different studied dyes (**b**).

As confirmed in the literature, the preparation of cellulosic composites with zinc oxide depends strongly on the methodology followed, which affects the morphology and structure of the ZnO particles [12] but also the existence of –OH groups available on the substrate surface that acts as a driving force during ZnO nucleation due to electrostatic interactions [28]. A mechanism of formation and action is described below (Equations (3)–(9) and Figure 7).

In general, as shown in Equation (3), the use of zinc acetate as a precursor for the growth of ZnO particles begins with the formation of Zn^2+^ ions, which interact strongly with the –OH present in the medium but also on the surface of the cellulose (Figure 7a). Thus, the formation of hydroxide Zn(OH)_2_ is forced [25], and when the medium is saturated with –OH groups of alkaline origin, the formation of Zn(OH)_4_^2−^ (Equation (4)) occurs on the surface and interstice of the fibers [26], which subsequently decomposes (Equation (5)), initiating the nucleation of ZnO [5].

(3) $${Zn}^{2+}+{2\;OH}^{-}\to {Zn(OH)}_{2}$$

(4) $${Zn(OH)}_{2}+{2\;OH}^{-}\to {Zn(OH)}_{4}^{2-}$$

(5) $${Zn(OH)}_{4}^{2-}\to ZnO+H_{2}O+{2\;OH}^{-}$$

Subsequently, the catalytic activity of ZnO is based on the creation of electron–hole pairs from the interaction with light (Equation (6)), achieving the generation of active species OH^•^ and O_2_^•^ in the aqueous media (Equations (7) and (8)) that interact with the pollutants [13,21] and degrade them (Figure 7b) into non-toxic and simpler compounds (Equation (9)). Thus, as previously discussed, oxygen vacancies could increase the affinity of ZnO for molecules such as O_2_ and H_2_O, promoting the formation of reactive species [20], such as OH^•^ and O_2_^•^, which are key in photocatalytic processes. Furthermore, it could be assumed that structural defects in ZnO would improve electron mobility in the crystal lattice, reducing recombination and increasing the efficiency of free radical generation.

(6) $$ZnO+h\nu \to ZnO\left(h^{+}+e^{-}\right)$$

(7) $${OH}^{-}+h^{+}\to {OH}^{•}$$

(8) $$O_{2}+e^{-}\to {O_{2}}^{•}$$

(9) $$dye+{OH}^{•}+{O_{2}}^{•}\to degradation\;products$$

To obtain the best comparative study, the photocatalytic activities of ZnO and cellulose/ZnO composites against various dyes were evaluated. It should be noted that although the dosage during these studies was 0.1 g of composite in each experiment, for ATcell/ZnO and FTcell/ZnO, approximately only 47% wt. of the sample corresponded to ZnO particles (as seen in Table 1). For this reason and in order to better evaluate the possible dual adsorptive/photocatalytic mechanism of the samples, the specific removal per ZnO active site (Rs, % removal/g ZnO) was defined as follows:

(10) $$Rs=\frac{R}{IM}\times 100$$

where R (%) is the dye removal achieved by tested mass g of the sample, calculated according to Equation (2), and IM (%) is the inorganic matter for the prepared material as calculated by Equation (1) and appeared in Table 1.

**Figure 8:**
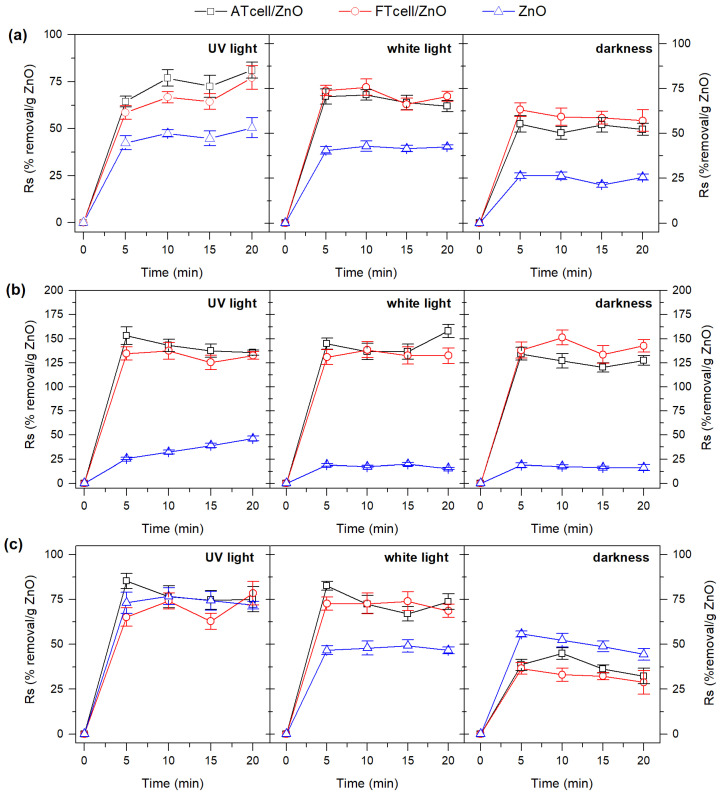
The performance of the ATcell/ZnO, FTcell/ZnO, and ZnO samples on the specific removal (Rs, % removal/ZnO content) of (**a**) MO, (**b**) MB, and (**c**) BB dyes.

As shown in Figure 8, there was a remarkable difference in dye removal efficiency across different light sources and composites. Notably, in most cases, the maximum dye degradation occurred within the first 5 min of exposure with the samples, without observing a progressive removal enhancement over time, which could suggest the saturation or inactivation of the catalyst surface by the dye molecule [30].

Figure 8a shows significant photocatalytic activity of ZnO on the removal of the MO dye under the conditions studied, as a maximum specific reduction of 51, 43, and 26% removal/g ZnO of the dye was achieved under UV light, white light, and no light irradiation. In the work of Rodwihok et al. [46], pristine ZnO particles (95 nm of crystal size) barely managed to adsorb MO in the dark (<5% removal) and reached approximately 20% of dye elimination after 20 min of sunlight irradiation. Zafar et al. [25] reported the adsorptive capacity of ZnO nanospheres prepared by the analogous alkaline precipitation method but with better textural properties (S_BET_ 49.36 m^2^ g^−1^ and D_BJH_ 27.44 nm) and where a dose of 0.1 g of nanoparticles managed to adsorb about 50% of MO under similar experimental conditions (pH 6 and room temperature). Thus, the importance of particle size on the adsorptive/photocatalytic removal capacity of ZnO resulted evident. The ATcell/ZnO and FTcell/ZnO cellulosic composites also showed certain yield sensitivity to light exposure, improving the specific MO removal capacity observed for pure ZnO by about 30% under all three experimental conditions. As discussed in the sample characterization, both cellulose/ZnO composites exhibited better textural (8–11% higher S_BET_ surface area and 5–13% lower average D_BJH_ pore size) and structural (56–63% reduction in D crystallite size) properties than the as-prepared ZnO particles. Thus, although the amount of zinc oxide present is lower in ATcell/ZnO and FTcell/ZnO, the improved surface area provided by the cellulose substrate for uniform and distributed particle growth had a tremendous positive impact on the MO dye removal capacity. By comparing the results of experiments carried out under UV light and those carried out in the dark, it can be confirmed that the removal of MO with the materials studied is effective following an adsorption mechanism and that it is enhanced with the application of white or ultraviolet light.

Similarly, for MB (cationic dye) removal assays, the adsorption/desorption behavior of the materials becomes a key factor, demonstrating the great influence of the catalysts surface on the MB dye removal efficiency. This is confirmed by the results shown in Figure 8b with a predominant photocatalytic effect of ZnO on the removal of MB, with a maximum around 15–17% of dye removal/ZnO content under white light or in the dark, while irradiation with UV light achieved close to 25% removal after 5 min of the experiment, increasing linearly up to 45% removal after a 20 min assay. The ATcell/ZnO and FTcell/ZnO composites showed good behavior in experiments conducted under three light conditions, removing near to 73% of the dye after 5 min of assay (with Rs up to 150%). As indicated by Dehghani et al. [37], the degradation of MB on cellulosic composites with ZnO requires a first adsorption step, allowing the contaminants to interact with the catalyst to facilitate the photodegradation process. These authors reported a maximum of 20% MB degradation after 20 min of solar or UV radiation using ZnO particles (12.15 m^2^/g surface area, 23 nm pore size), while when supported on cellulose nanofibers (with a relative ZnO loading of 29–72% wt.), they achieved 30–95% degradation. For ZnO concentrations similar to those found in ATcell/ZnO and FTcell/ZnO (around 47% wt according to Table 1), the aforementioned work [37] achieved less than 40% MB removal in 20 min (using IZnOCNF sample with 2.24 m^2^/g surface area and 63 nm pore diameter) but over 80% degradation with a composite with better textural properties (using VIZnOCNF with 10.94 m^2^/g and 7.9 nm pore diameter). In fact, adsorption proved to be the main mechanism for this dye removal by the ATcell/ZnO and FTcell/ZnO samples, as seen in Figure 8b (experiments irradiated with light or in the dark).

Again, the dual adsorptive/photocatalytic effect of ZnO was also clear, as shown in Figure 8c, where a better adsorption of BB on the ZnO surface could be observed (between 44 and 47% Rs under white light or in darkness after 20 min), promoted by the possible ionization of Zn-OH groups on the catalyst surface, which also achieves a degradation of about 72% Rs of BB after 20 min when the experiment is irradiated with UV light. There are few studies reporting results on the potential adsorption and photodegradation of BB from aqueous effluents [29]. The rapid and complete removal of BB (12 min under visible light) has been reported [47] using a combined system of a recoverable biogenic hematite photocatalyst (G-Fe_2_O_3_-NP’s) with the oxidizing agent H_2_O_2_. Another example is the work of Akpomie et al. [48], where only the adsorptive capacity of ZnO nanoparticles was evaluated under different conditions (pH, dye concentration, ZnO dosage, temperature, and previous sonication). Thus, in similar experiments, these authors found a 50–70% removal of BB after 180 min of testing using loads of 0.1–0.3 g of ZnO nanoparticles, dropping to approximately 20% removal in 20 min. In the present work, the ATcell/ZnO and FTcell/ZnO composites achieved a net adsorption (dark tests) of 13–15% of the dye (Rs of 28–32% removal/g ZnO in Figure 8c), improving to up to 32–37% removal after 20 min under UV and white light irradiation (Rs of 69–79% BB removal/ZnO content in the sample). Lower yields of 20% in BB photodegradation have been reported by Shah et al. [49] after 2 h of UV light exposure using graphene nanoplate-supported titanium oxide (TiO_2_/GNP). However, no references have been found to studies on the adsorption/photodegradation processes for BB using cellulose composites with zinc oxide, the present work being the first to report results.

**Figure 9:**
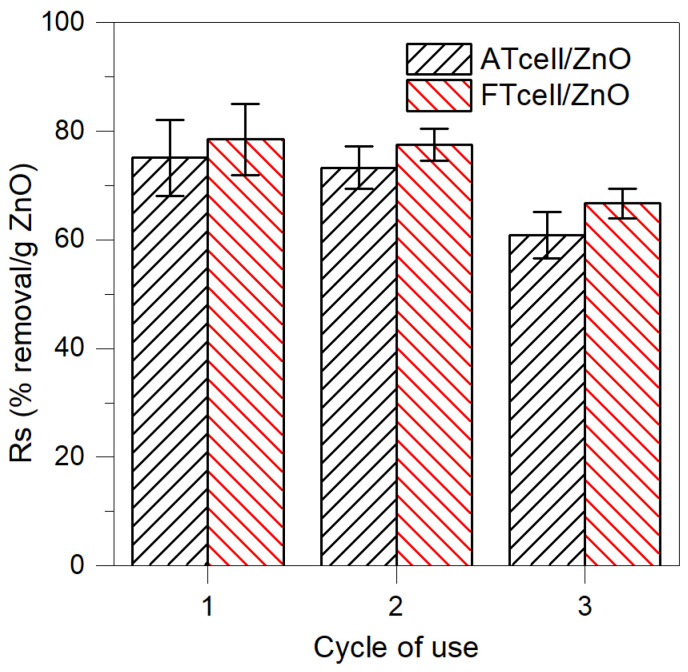
Specific removal (Rs, % removal/ZnO content) reached by regenerated and reused ATcell/ZnO and FTcell/ZnO composites for BB removal experiments (20 min contact under UV light irradiation).

The reusability study was performed for the BB removal process irradiated with UV light. As shown in Figure 9, both composites showed adequate behavior in the second dye removal cycle, with the Rs value slightly reducing from 75.11 ± 6.99% to 73.28 ± 3.92% for the ATcell/ZnO composite and from 78.47 ± 6.55% to 77.45 ± 2.96% when FTcell/ZnO was used as the photo-adsorbent agent.

After the third reuse, the efficiency decreased slightly for both samples, resulting in Rs values of 60.83–4.30% and 66.70–2.76% for ATcell/ZnO and FTcell/ZnO, respectively; that is, after three uses, the composites lost 19% and 15% of their initial capacity to remove BB dye under UV light. Other authors did not observe loss of stability or activity of their cellulosic composites with ZnO [26], while others found only a 10% loss of efficacy after 5 reuse cycles [13]. The reusability of this type of hybrid material with photo-adsorptive capacity is very important for its application in wastewater treatment processes, and it is considered necessary to study various eluents that can regenerate the composites without affecting their stability and effectiveness [29,39].

### 3.3. Understanding the Effect of Cellulose/ZnO Composite Properties in Dye Removal Success

**Figure 10:**
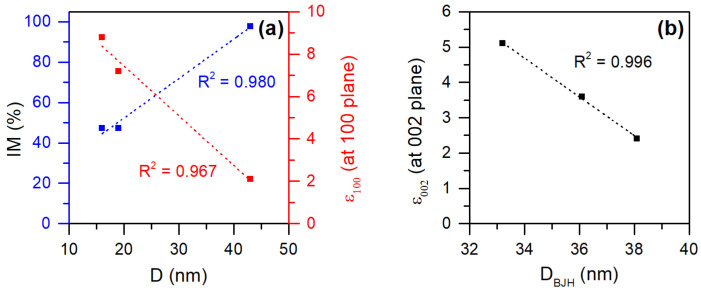
Linear fit analyses for parameter pairs. Relationship between compositional and structural characteristics on the crystallite size D of the grown ZnO particles (**a**) and on the average pore diameter D_BJH_ of the prepared samples (**b**).

The multivariate statistical analysis performed on the set of results obtained (Tables S3–S5 in SI) allowed us to distinguish significant differences in the behavior of the materials during the removal of the three dyes considered. In this way, it was possible to verify that the structural and textural properties evaluated for the tested materials showed a significant relationship with each other, as shown in Figure 10.

Figure 10a shows the most significative relationships found, with ZnO crystallite size (D) strongly and directly related with the inorganic load (IM) present in ZnO, ATcell/ZnO, and FTcell/ZnO samples (see Table 1), avoiding ZnO particle agglomeration when cellulose was used as support. An indirect relationship of crystallite size was also observed with the lattice strain at the 100 plane (Table S2 in SI). As discussed in the literature [28], the 100 plane of ZnO has a higher surface energy than others, and it is in this plane where there is greater interaction between ZnO and cellulose [33], avoiding particle growth in the direction of the c plane and limiting their size. Thus, the higher the ε_100_, the smaller the diameter D of the ZnO crystals formed. These authors also indicated that the electrostatic interactions between ZnO and cellulose, which cause a slower growth of the particles, force the development along the surface of the cellulose, affecting the 002 plane of the zinc oxide, thus favoring a uniform superficial growth, affecting the textural properties of the material. This can be seen in Figure 10b, where the strong relationship between the lattice strain ε_002_ and the average pore diameter D_BJH_ is observed, such that the higher tensions in the formed crystals translate into a less compacted and dispersed growth, improving the porosity of the surface.

**Figure 11:**
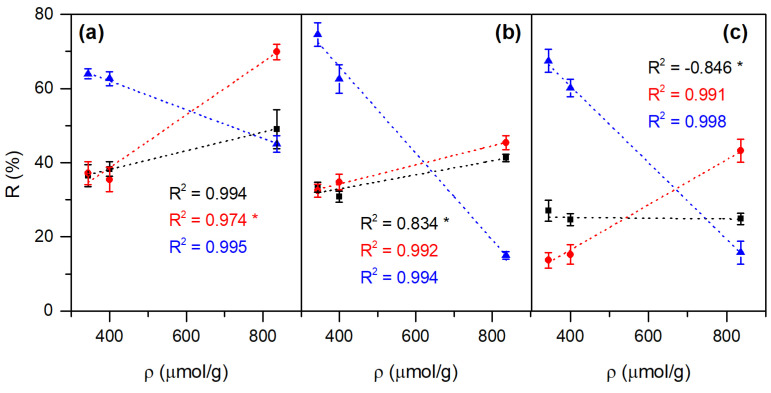
Fitted curves of removal extent (R, %) and surface charge of the prepared materials (ρ, μmol/g) after 20 min exposure with MO (black square), BB (red dot), or MB (blue triangle) under UV light (**a**), white light (**b**), or in darkness (**c**). For each curve, the adjusted R^2^ is presented and * denotes that the slope is NOT significantly dependent on data at 95% confidence (*p* > 0.05).

The combined multivariable analysis of all experiments showed no significant effect of evaluated properties on elimination extent, so an individual study was conducted for each dye. Interestingly, surface charge ρ was strongly associated with the extent of pollutant removal from the experiments performed, and more specifically with the type of dye, as shown in the fitted curve graphs in Figure 11. It can be seen how the presence of positive charges, due to the protonation of the hydroxyl groups present on the surface of ZnO, strongly influences any type of applied light, which clearly makes the surface charge a key factor in the removal mechanisms. Furthermore, while for MO and BB, the R-ρ relationship is direct, for MB, it is strongly indirect, which is confirmed by the cationic nature of this dye and the stronger repulsive interactions with the surface of the ZnO composite [29]. Under UV light irradiation (Figure 11a), the removal efficiency was greater for BB (red dot curve), a weakly charged molecule in its anionic form that would have a sulfonate group and an alkoxide in its structure (Table S1), while MO (black square curve) only has one sulfonate group, so its possible interaction with the surface of the composite is reduced (less steep slope in the fitted curve). In addition, BB is known to absorb in the UV light range, making the removal observed for this dye more pronounced due to the photocatalytic nature of the process.

When experiments were carried out under white light (Figure 11b), MO experienced almost the same degradation as under UV radiation, and the removal performance for BB was comparable to the MO one, both dyes being of anionic character. The MB retained a strong indirect relationship between removal and the surface charge of the composite used, but under white light, this relationship was even stronger (steeper curve slope), suggesting that the adsorption of the dye on the surface of the ZnO-prepared materials was better. Likewise, MB removal in darkness (blue triangle in Figure 11c) followed the same behavior as under visible light, obeying mainly an adsorption mechanism highly influenced by the surface charge of the material, being much better for prepared cellulose/ZnO composites than for pristine ZnO particles. While for MO almost no influence of the surface charge of the material on the removal success was distinguished, the BB dye experienced the lowest removal values in darkness, although directly dependent on the positive charge of the adsorbent surface, which clarifies the potential combined use of photocatalysts/adsorbents in processes for the elimination of this pollutant, which has not been studied to date and opens an interesting research scope for the application of hybrid cellulosic materials with photocatalytic properties.

## 4. Conclusions

In this study, ZnO nanoparticles were first synthesized onto the cellulose fibers surface using a chemical in situ method. The prepared samples were subsequently characterized, demonstrating a significant improvement in cellulose surface texture thanks to the presence of ZnO particles (up to eight-fold higher S_BET_ and up to 60% reduction in D_BJH_ than the initial cellulose) as well as the appearance of optical properties similar to those of ZnO (bandgap close to 3.2 eV and performance in the UV and visible range). In this way, it was possible to confirm that the prepared composites could have potential applications as adsorbents/photocatalysts. Both cellulose/ZnO composites demonstrated good performance for specific removal of MO, MB, or BB pollutants, driven by the synergistic effects of adsorptive and photocatalytic mechanisms. Thus, the ATcell/ZnO and FTcell/ZnO composites showed a preferential MB removal mechanism by adsorption (up to 75% in 20 min), mainly caused by electrostatic attraction between the dye and the adsorbent surface. For the anionic dyes MO and BB, the dual adsorption/photodegradation performance of the prepared composites had evident results, with different behaviors observed depending on the type of light applied. This demonstrated how the use of a suitable support, such as cellulose, can significantly improve the dispersion of ZnO particles (smaller particles and prevent agglomeration), with clear improvements in their photocatalytic activity and surface properties. The obtained results for MO removal were similar to some found in the literature (with approximately 40% removal after 20 min of UV light). However, no references have been found to studies on the adsorption/photodegradation processes for BB using cellulose composites with zinc oxide, the present work being the first to report some results (about 35% elimination after 20 min of white or UV light). This study highlights the potential of cellulose/ZnO composites as highly effective photocatalysts for wastewater treatment in the textile industry. Future research could focus on the comparison of the composites’ formulation, reusability, and testing effectiveness against other dye and organic pollutants to further advance environmental remediation.

## Figures and Tables

**Table 1:** Some chemical, morphological, and structural properties of cellulosic samples (fiber length L, fiber width W, inorganic content IM, surface charge ρ, crystalline index CrI, surface area S_BET_, and average pore size D_BJH_).

Sample	L ^a^ (mm)	W ^a^ (μm)	IM ^b^ (%)	ρ ^c^ (μmol/g)	CrI ^d^ (%)	S_BET_ ^e^ (m^2^/g)	D_BJH_ ^e^ (Å)
ATcell	0.57 ± 0.04	19.3 ± 0.6	1.3 ± 0.2	51.0 ± 1.0	66.34	1.80	798
FTcell	0.54 ± 0.03	22.7 ± 0.3	2.0 ± 0.8	79.81 ± 1.7	66.42	3.32	563
ATcell/ZnO	n.d	n.d	47.2 ± 0.8	399.9 ± 10.1	49.68	14.48	332
FTcell/ZnO	n.d	n.d	47.3 ± 0.7	343.7 ± 21.1	52.21	14.94	361
ZnO	n.d	n.d	97.2 ± 0.4	836.2 ± 21.1	n.d	13.42	381

(n.d) not determined for this sample; (a) determined by MORFI analysis; (b) gravimetrically determined according to Equation (1); (c) determined by Equation (S1) after conductometric titration; (d) determined for cellulosic materials by Equation (S2) from XRD analyses; (e) determined by N_2_ adsorption/desorption porosimetry.

## Data Availability

The original contributions presented in this study are included in the article/Supplementary Material. Further inquiries can be directed to the corresponding author.

## Supplementary Materials

The following supporting information can be downloaded at: https://www.mdpi.com/article/10.3390/polym17131737/s1, Table S1: Dyes used for the evaluation of adsorptive/photocatalytic efficiency of cellulose/ZnO composites; Text S1: Characterization methods; Text S2. Removal of dyes under controlled light irradiation; Figure S1: Scheme of the photoreactor prototype used in this work (a) and recorded spectra for the employed UV (b) and white (c) light sources; Figure S2: Optical Microscopy observations of ZnO particles and cell/ZnO composites; Table S2: X-ray diffraction planes considered during crystallite size (D, nm) and lattice strain (ε) determination for ZnO particles present in the prepared samples; Text S3. Statistical analysis; Table S3: Statistically determined correlation parameters for variables considered during methyl orange (MO) removal; Table S4: Statistically determined correlation parameters for variables considered during methylene blue (MB) removal; Table S5: Statistically determined correlation parameters for variables considered during bromophenol blue (BB) removal. References [35,36] are cited in the Supplementary materials.

## Abbreviations

The following abbreviations are used in this manuscript:

AT	Almond tree
ATcell	Cellulose extracted from almond tree
BB	Bromophenol blue
CrI	Crystalline index (%)
D	Crystallite size (nm)
D_BJH_	average pore diameter ()
Eg	Bandgap (eV)
FT	Fig tree
FTcell	Cellulose extracted from fig tree
IM	Inorganic matter (% wt.)
L	Fiber length (mm)
MB	Methylene blue
MO	Methyl orange
R	Dye removal (%)
Rs	Specific dye removal (% removal/g ZnO)
S_BET_	Surface area (m^2^/g)
W	Fiber width (μm)
ε_002_	Lattice strain at 002 plane in ZnO structure
ε_100_	Lattice strain at 100 plane in ZnO structure
ρ	Surface charge (μmol/g)
