# Supplementary material for: Agricultural Waste-Derived Cellulose/ZnO Composites: Dual Photocatalytic and Adsorptive Action for Textile Dye Removal
Source: Polymers (Basel). 2025 Jun 22;17(13):1737. doi: 10.3390/polym17131737 (PMC12252186; doi:10.3390/polym17131737)
Supplement: Supplementary file 1 [file polymers-17-01737-s001.zip › polymers-3641650-supplementary.pdf]

SUPPLEMENTARY INFORMATION

Agricultural Waste-Derived Cellulose/ZnO Composites: Dual Photocatalytic and Adsorptive Action for Textile Dye Removal

Jihene Belhaj[1,2], Ramzi Khiari[3,4], Valentín García-Caballero[5], Antonio A. Romero[1,6], Araceli Garcia[1,6,*]

[1] Nanoval FQM-383 Research Group, Organic Chemistry Department, University of Córdoba, Marie Curie (C-3) Building, Crta. Nnal. km 396, E-14014 Córdoba, Spain

[2] National Engineering School of Monastir, University of Monastir, Monastir 5019, Tunisia

[3] Department of Textile, Higher Institute of Technological Studies of Ksar Hellal, Ksar Hellal 5070, Tunisia

[4] CNRS, Grenoble INP, LGP2, Université Grenoble Alpes, F-38000 Grenoble, France

[5] Departamento de Química Física y Termodinámica Aplicada, Instituto Químico para la Energía y el Medioambiente (IQUEMA), Universidad de Córdoba, E-14014 Córdoba, Spain

[6] Instituto Químico Para la Energía y el Medioambiente (IQUEMA), Faculty of Sciences, University of Córdoba, Marie Curie (C-3) Building, Crta. Nnal. km 396, E-14014 Córdoba, Spain

* Corresponding author: qo2ganua@uco.es

SI index

Table S1. Dyes used for the evaluation of the adsorptive/photocatalytic efficiency of cellulose/ZnO composites.

Text S1. Characterization methods.

Text S2. Removal of dyes under controlled light irradiation.

Figure S1. The scheme of the photoreactor prototype used in this work (a) and recorded spectra for the employed UV (b) and white (c) light sources.

Figure S2. Optical Microscopy observations of ZnO particles and cell/ZnO composites.

Table S2. X-ray diffraction planes considered during crystallite size (D, nm) and lattice strain (ε) determination for ZnO particles present in the prepared samples.

Text S3. Statistical analysis.

Table S3. Statistically determined correlation parameters for variables considered during methyl orange (MO) removal.

Table S4. Statistically determined correlation parameters for variables considered during methylene blue (MB) removal.

Table S5. Statistically determined correlation parameters for variables considered during bromophenol blue (BB) removal.

Table S1. Dyes used for the evaluation of the adsorptive/photocatalytic efficiency of cellulose/ZnO composites.

**Dye**	**Methyl Orange (MO)**	**Methylene Blue (MB)**	**Bromophenol Blue (BB)**
Structure	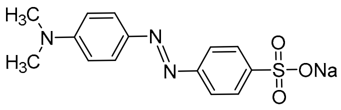	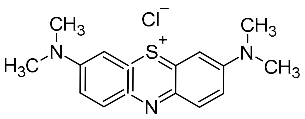	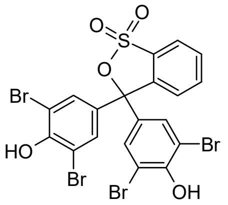
Formula	$C_{16}H_{18}ClN_3S$	$C_{14}H_{14}N_3NaO_3S$	$C_{19}H_{10}Br_4O_5S$
Mw (g/mol)	319.85	327.33	669.96
λ max (nm)	464	664	598
Use (type)	Dye (anionic)	Dye (cationic)	Dye (anionic)

Table S1 shows for each of the three dyes studied in the present work the chemical structure, the molecular weight (Mw g/mol), the wavelength at which each dye presents maximum absorption (λ nm), and the type or nature of each one.

Text S1. Characterization methods.

FT-IR analyses of cellulose and cellulose/ZnO composites were conducted using an FTIR-ATR Spectrum Two (Perkin Elmer, USA) spectrophotometer with a resolution of 4 cm^{-1} and 20 scans in the range of 500 to 4000 cm^{-1}. The FT-IR spectra were processed and then compared in order to evaluate the effect of ZnO incorporation into the cellulose from different vegetal sources.

ZnO and cellulose/ZnO composites were characterized using optical microscopy, scanning electron microscopy (MEB FEG ZEISS Ultra 55), and energy dispersive X-ray spectroscopy (SEM-EDX). Microscopy images were obtained using a Carl Zeiss Axio Imager M1 optical microscope equipped with a digital camera. The examined sample suspensions were diluted, and a droplet was placed between the glass slide and a coverslip. The images were captured by an AxioCam MRc 5 digital camera. The SEM-EDX analysis involved scanning the surface of a sample with a focused beam of electrons accelerated to voltages between 5 and 30 kV. These electrons interact with the sample's surface. Each sample was metallized with a gold/palladium coating before analysis. Additionally, SEM-EDX spectra were collected for each image.

The surface chemical information of the samples was determined and recorded by X-ray photoelectron spectra (XPS) in a spectrometer Phoibos 150 MCD (Specs, Germany), with a MgKα source (1253.6 eV) in a “stop-and-go” mode to reduce potential damage due to sample irradiation and using C1s as a reference (284.6 eV). Powdered samples were deposited on a sample holder and then evacuated under vacuum ($<10^{-6}$ Torr).

UV-vis diffuse reflectance spectroscopy (DRS) was performed in a Lambda 650 S UV-Vis spectrometer (PerkinElmer, USA) recording the absorbance between 250 and 800 nm. Automatically generated Kubelka–Munk function plots were used to determine the band gap energy (Eg, eV) for each sample.

The surface charge of each sample was determined by conductimetric titration [1], [2] with a 0.01 $mol \cdot L^{-1}$ NaOH solution using an PC80 pH/mV/Cond/Temp Bench Meter (XS® Instruments, Italy). Briefly, 150 mg of the sample was added into 60 mL of ultrapure water. After 1 h under constant stirring, the suspension was titrated, and the conductivity was plotted

against added volume of NaOH solution for surface charge ρ (mmol/g) determination with equation (S1).

$$\rho = \frac{C_{NaOH} \times V_{NaOH}}{m_s} \quad \text{(S1)}$$

where C_{NaOH} is the concentration ($mol \cdot L^{-1}$) of the standardized NaOH solution, V_{NaOH} is the consumed volume (mL) of NaOH solution at the inflection point [3], and m_s (g) is the mass of tested sample. This measurement was performed in duplicate for each sample.

X-ray diffraction (XRD) analyses were conducted in order to check structure and crystallinity variation of the cellulose fibers and the prepared cellulose/ZnO composites. XRD measurements were performed in a Bruker D8 DISCOVER (Bruker, USA) using Cu Kα radiation (1.54056 λ, 40 kV, 40 mA). All samples were scanned with a step size of 0.02° and a count time of 0.1 s per step over the 2θ range 5–100°. The crystalline index (CrI, %) of each cellulosic sample was determined according to the following Segal equation (S2):

$$CrI(\%) = \frac{(I_{200} - I_{am})}{I_{200}} \times 100 \quad \text{(S2)}$$

where I_{200} represents the maximum intensity of the (200) lattice peak and I_{am} represents the intensity of the amorphous region at approximately 2θ =18° for cellulosic samples. The Debye–Scherrer theory was used to determine the crystallite size (D, nm) (equation S3) and the approximation of the lattice strain (ε) in the ZnO structures (equation S4), which were formed for the different samples [4], [5].

$$D(nm) = \frac{0.89 \times \lambda}{\beta \times \cos\theta} \quad \text{(S3)}$$

$$\varepsilon = \frac{\beta}{4 \times \tan\theta} \quad \text{(S4)}$$

where λ was the wavelength used during the X-ray measurements (λ = 1.15046 nm), β is the full width in radian at half-maximum of the diffraction peak, and θ is the Bragg angle of the XRD pattern [6]. These two crystallite parameters were measured at different planes.

The textural properties of ZnO and cellulosic samples were analyzed using nitrogen adsorption/desorption measurements in an ASAP 2000 porosimeter (Micromeritics Instrument Corp., USA) with a degasification temperature of 100 °C for 24 h. The surface area (S_{BET}) was calculated using the linear part (0.05 < Po < 0.30) of the Brunauer, Emmett, and Teller (BET) equation. Pore size distribution and pore volume were estimated using the Barrett, Joyner, and Halenda (BJH) equation.

Mott–Schottky analyses of different samples were carried out in a nitrogen-saturated atmosphere. Electrochemical measurements were performed in a classical three-electrode cell, using a BioLogic VMP-3e potentiostat/galvanostat. An amount of 0.1 M KOH solution was used as an aqueous electrolyte solution. Ag/AgCl and graphite rods were used as a reference and counter electrodes, respectively. A glassy carbon disk (GCE, 5 mm in diameter, from Pine Research, Ref. AFE3T050GC) was used as a working electrode. The mass loading was constant for all the samples. All working electrodes were modified with the different materials by drop-casting of 25 μL of the sample at a concentration of 5 mg·mL^{-1}. All potentials were referenced to the reversible hydrogen electrode (RHE) using the Nernst equation [7].

Text S2. Removal of dyes under controlled light irradiation.

The dye removal experiments were conducted in a handmade photoreactor prototype (**Figure S1a)** consisting of a cardboard cylinder, internally lined with LEDs strip, with a glass tube supported inside where the reaction, assisted by magnetic stirring, occurred at room temperature. The LED strips were selected as UV light or white light source, whose light intensity was determined using a portable LED meter MK350S (UPRtek, Taiwan) at the beginning of each experiment to ensure quality and replicability of the obtained results.

Figures S1b and S1c show the measurement LED meter reports for the prototype using UV LEDs (395-400 nm, PEREL Vellemangroup Belgium) and white light LEDs (RGB, ELECTRO DH Spain), respectively, with λ (nm) on X-axis and normalized intensity on Y-axis. For UV light (Figure S1b), a maximum intensity was recorded at 399 nm, whereas for white light, the maximum intensity occurred at 465 nm (blue), with another two peaks at 523 nm (green) and at 635 nm (red) wavelengths.

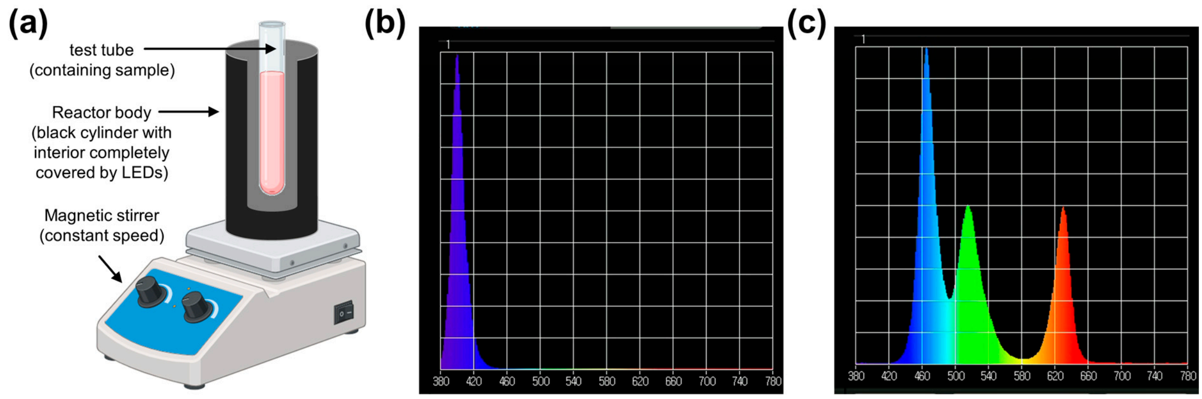

Figure S1. The scheme of the photoreactor prototype used in this work (a) and recorded spectra for the employed UV (b) and white (c) light sources.

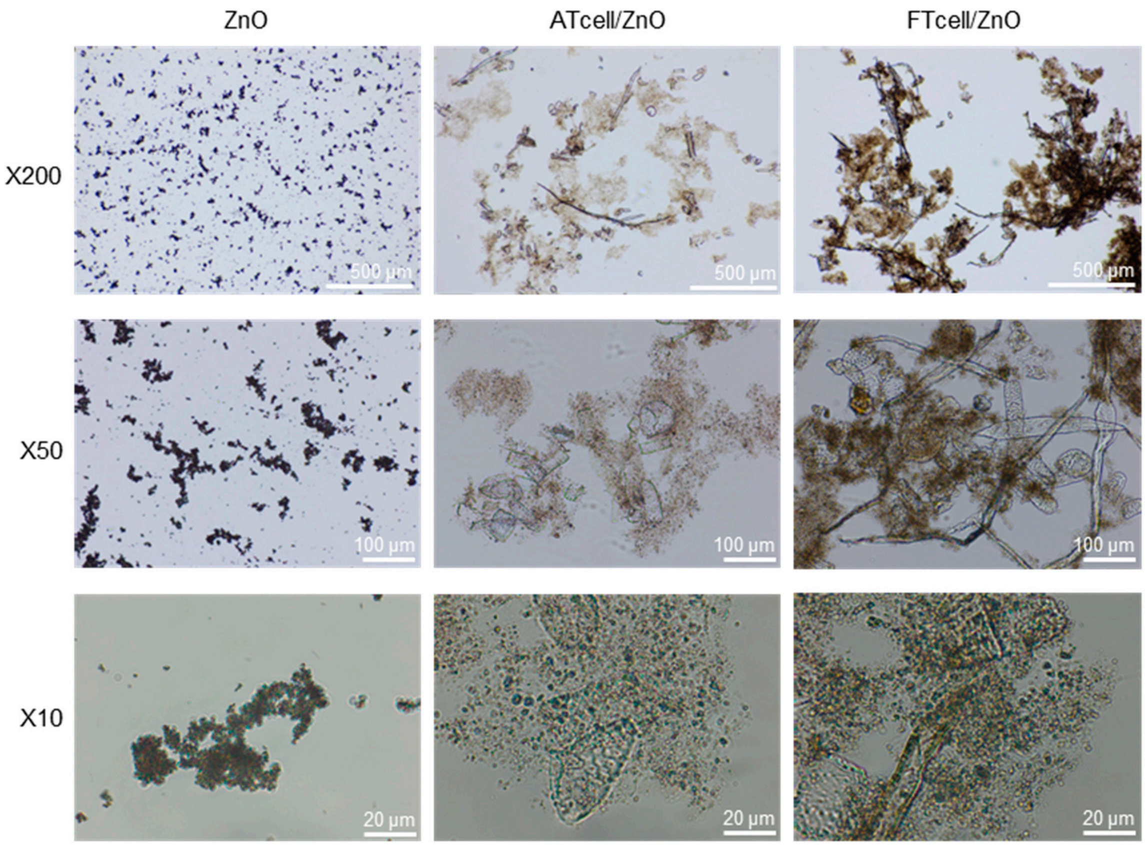

Figure S2. Optical Microscopy observations of ZnO particles and cell/ZnO composites.

Table S2. X-ray diffraction planes considered during crystallite size (D, nm) and lattice strain (ε) determination for ZnO particles present in the prepared samples.

Sample	Parameter	Planes							
		(100)	(002)	(101)	(102)	(110)	(103)	(112)	(201)
	2θ	31.68	34.32	36.15	47.41	56.46	62.71	67.79	68.93
ZnO	D (nm)	62.8	52.2	53.9	36.5	35.6	34.4	34.4	32.3
	ε	$2.1 \cdot 10^{-3}$	$2.4 \cdot 10^{-3}$	$2.2 \cdot 10^{-3}$	$2.5 \cdot 10^{-3}$	$2.2 \cdot 10^{-3}$	$2.0 \cdot 10^{-3}$	$1.8 \cdot 10^{-3}$	$2.0 \cdot 10^{-3}$
	2θ	31.44	34.10	35.94	47.25	56.28	62.60	67.74	68.81
ATcell/ZnO	D (nm)	15.2	24.2	17.1	14.2	16.6	16.6	14.0	14.1
	ε	$8.8 \cdot 10^{-3}$	$5.1 \cdot 10^{-3}$	$6.9 \cdot 10^{-3}$	$6.4 \cdot 10^{-3}$	$4.6 \cdot 10^{-3}$	$4.2 \cdot 10^{-3}$	$4.7 \cdot 10^{-3}$	$4.6 \cdot 10^{-3}$
	2θ	31.42	34.08	35.92	47.21	56.28	62.60	67.64	68.87
FTcell/ZnO	D (nm)	18.5	34.8	20.9	14.8	18.1	16.9	16.1	13.6
	ε	$7.2 \cdot 10^{-3}$	$3.6 \cdot 10^{-3}$	$5.6 \cdot 10^{-3}$	$6.1 \cdot 10^{-3}$	$4.2 \cdot 10^{-3}$	$4.1 \cdot 10^{-3}$	$4.1 \cdot 10^{-3}$	$4.7 \cdot 10^{-3}$

Table S2 details the different crystallographic planes representing the wurtzite structure of pristine ZnO or ZnO grown on the surface of ATcell or FTcell celluloses, forming their corresponding cellulose/ZnO composites. The crystallite sizes calculated for each plane are also shown, allowing us to verify that the ZnO crystallites grown on cellulose showed a clearly smaller and more regular size than pristine ZnO. Regarding the lattice strain, the values calculated for the ATcell/ZnO and FTcell/ZnO samples indicated an increase in the stress/strain of the crystallites in all their planes, which could indicate a strong interaction with the cellulose surface where they were grown [8].

Text S3. Statistical analysis.

Tables S3 to **S5** shows Pearson product moment correlations between each pair of studied variables from the three different dye removal experiments performed. These correlation coefficients range between -1 and +1 and measure the strength of the linear relationship between the variables. Also shown in parentheses is the number of pairs of data values used to compute each coefficient. The third number in each location of the table is a P-value, which tests the statistical significance of the estimated correlations. P-values below 0.05 (red highlighted) indicate statistically significant non-zero correlations at the 95.0% confidence level.

Table S3. Statistically determined correlation parameters for variables considered during methyl orange (MO) removal.

	**R**	**SAMPLE**	**IM**	**ρ**	**D**	**ε**	**S_{BET}**	**D_{BJH}**	**E_g**
**R**		-0.380<br>0.314	0.654<br>0.056	0.611<br>0.081	0.688<br>0.041	-0.750<br>0.020	-0.535<br>0.137	0.748<br>0.021	0.295<br>0.441
**SAMPLE**	-0.380<br>0.314		-0.865<br>0.003	-0.913<br>0.001	-0.811<br>0.008	0.444<br>0.232	0.966<br>0.000	-0.406<br>0.278	0.596<br>0.090
**IM**	0.654<br>0.056	-0.865<br>0.003		0.994<br>0.000	0.995<br>0.000	-0.833<br>0.005	-0.966<br>0.000	0.810<br>0.008	-0.113<br>0.772
**ρ**	0.611<br>0.081	-0.913<br>0.001	0.994<br>0.000		0.979<br>0.000	-0.770<br>0.015	-0.988<br>0.000	0.743<br>0.022	-0.218<br>0.574
**D**	0.688<br>0.041	-0.811<br>0.008	0.995<br>0.000	0.979<br>0.000		-0.884<br>0.002	-0.935<br>0.000	0.864<br>0.003	-0.013<br>0.973
**ε**	-0.750<br>0.020	0.444<br>0.232	-0.833<br>0.005	-0.770<br>0.015	-0.884<br>0.002		0.661<br>0.052	-0.999<br>0.000	-0.455<br>0.218
**S_{BET}**	-0.535<br>0.137	0.966<br>0.000	-0.966<br>0.000	-0.988<br>0.000	-0.935<br>0.000	0.661<br>0.052		-0.630<br>0.069	0.367<br>0.332
**D_{BJH}**	0.748<br>0.021	-0.406<br>0.278	0.810<br>0.008	0.743<br>0.022	0.864<br>0.003	-0.999<br>0.000	-0.630<br>0.069		0.492<br>0.179
**E_g**	0.295<br>0.441	0.596<br>0.090	-0.113<br>0.772	-0.218<br>0.574	-0.013<br>0.973	-0.455<br>0.218	0.367<br>0.332	0.492<br>0.179	

Table S4. Statistically determined correlation parameters for variables considered during methylene blue (MB) removal.

	**R**	**SAMPLE**	**IM**	**ρ**	**D**	**ε**	S_{BET}	D_{BJH}	E_g
**R**		0.850<br>0.004	-0.903<br>0.001	-0.912<br>0.001	-0.885<br>0.002	0.677<br>0.045	0.907<br>0.001	-0.651<br>0.058	0.238<br>0.538
**SAMPLE**	0.850<br>0.004		-0.865<br>0.003	-0.913<br>0.001	-0.811<br>0.008	0.444<br>0.232	0.966<br>0.000	-0.406<br>0.278	0.596<br>0.090
**IM**	-0.903<br>0.001	-0.865<br>0.003		0.994<br>0.000	0.995<br>0.000	-0.833<br>0.005	-0.966<br>0.000	0.810<br>0.008	-0.113<br>0.772
**ρ**	-0.912<br>0.001	-0.913<br>0.001	0.994<br>0.000		0.979<br>0.000	-0.770<br>0.015	-0.988<br>0.000	0.743<br>0.022	-0.218<br>0.574
**D**	-0.885<br>0.002	-0.811<br>0.008	0.995<br>0.000	0.979<br>0.000		-0.884<br>0.002	-0.935<br>0.000	0.864<br>0.003	-0.013<br>0.973
**ε**	0.677<br>0.045	0.444<br>0.232	-0.833<br>0.005	-0.770<br>0.015	-0.884<br>0.002		0.661<br>0.052	-0.999<br>0.000	-0.455<br>0.218
S_{BET}	0.907<br>0.001	0.966<br>0.000	-0.966<br>0.000	-0.988<br>0.000	-0.935<br>0.000	0.661<br>0.052		-0.630<br>0.069	0.367<br>0.332
D_{BJH}	-0.651<br>0.058	-0.406<br>0.278	0.810<br>0.008	0.743<br>0.022	0.864<br>0.003	-0.999<br>0.000	-0.630<br>0.069		0.492<br>0.179
E_g	0.238<br>0.538	0.596<br>0.090	-0.113<br>0.772	-0.218<br>0.574	-0.013<br>0.973	-0.455<br>0.218	0.367<br>0.332	0.492<br>0.179	

Table S5. Statistically determined correlation parameters for variables considered during bromophenol blue (BB) removal.

	**R**	**SAMPLE**	**IM**	**ρ**	**D**	**ε**	**S_{BET}**	**D_{BJH}**	**E_g**
**R**		-0.650<br>0.058	0.740<br>0.023	0.738<br>0.023	0.734<br>0.024	-0.605<br>0.085	-0.720<br>0.029	0.586<br>0.097	-0.104<br>0.789
**SAMPLE**	-0.650<br>0.058		-0.865<br>0.003	-0.913<br>0.001	-0.811<br>0.008	0.444<br>0.232	0.966<br>0.000	-0.406<br>0.278	0.596<br>0.090
**IM**	0.740<br>0.023	-0.865<br>0.003		0.994<br>0.000	0.995<br>0.000	-0.833<br>0.005	-0.966<br>0.000	0.810<br>0.008	-0.113<br>0.772
**ρ**	0.738<br>0.023	-0.913<br>0.001	0.994<br>0.000		0.979<br>0.000	-0.770<br>0.015	-0.988<br>0.000	0.743<br>0.022	-0.218<br>0.574
**D**	0.734<br>0.024	-0.811<br>0.008	0.995<br>0.000	0.979<br>0.000		-0.884<br>0.002	-0.935<br>0.000	0.864<br>0.003	-0.013<br>0.973
**ε**	-0.605<br>0.085	0.444<br>0.232	-0.833<br>0.005	-0.770<br>0.015	-0.884<br>0.002		0.661<br>0.052	-0.999<br>0.000	-0.455<br>0.218
**S_{BET}**	-0.720<br>0.029	0.966<br>0.000	-0.966<br>0.000	-0.988<br>0.000	-0.935<br>0.000	0.661<br>0.052		-0.630<br>0.069	0.367<br>0.332
**D_{BJH}**	0.586<br>0.097	-0.406<br>0.278	0.810<br>0.008	0.743<br>0.022	0.864<br>0.003	-0.999<br>0.000	-0.630<br>0.069		0.492<br>0.179
**E_g**	-0.104<br>0.789	0.596<br>0.090	-0.113<br>0.772	-0.218<br>0.574	-0.013<br>0.973	-0.455<br>0.218	0.367<br>0.332	0.492<br>0.179	

References

[1] S. Beck, M. Méthot, and J. Bouchard, ‘General procedure for determining cellulose nanocrystal sulfate half-ester content by conductometric titration’, *Cellulose*, vol. 22, no. 1, pp. 101–116, Feb. 2015, doi: 10.1007/s10570-014-0513-y.

[2] D. Musino, C. Rivard, G. Landrot, B. Novales, T. Rabilloud, and I. Capron, ‘Hydroxyl groups on cellulose nanocrystal surfaces form nucleation points for silver nanoparticles of varying shapes and sizes’, *Journal of Colloid and Interface Science*, vol. 584, pp. 360–371, Feb. 2021, doi: 10.1016/j.jcis.2020.09.082.

[3] A. García, J. Labidi, M. N. Belgacem, and J. Bras, ‘The nanocellulose biorefinery: woody versus herbaceous agricultural wastes for NCC production’, *Cellulose*, vol. 24, no. 2, pp. 693–704, Feb. 2017, doi: 10.1007/s10570-016-1144-2.

[4] X. Li, L. Zhang, Z. Wang, S. Wu, and J. Ma, ‘Cellulose controlled zinc oxide nanoparticles with adjustable morphology and their photocatalytic performances’, *Carbohydrate Polymers*, vol. 259, p. 117752, May 2021, doi: 10.1016/j.carbpol.2021.117752.

[5] A. A. Badawy, A. F. Ghanem, M. A. Yassin, A. M. Youssef, and M. H. Abdel Rehim, ‘Utilization and characterization of cellulose nanocrystals decorated with silver and zinc oxide nanoparticles for removal of lead ion from wastewater’, *Environmental Nanotechnology, Monitoring & Management*, vol. 16, p. 100501, Dec. 2021, doi: 10.1016/j.enmm.2021.100501.

[6] H. Li *et al.*, ‘Macro-/nanoporous Al-doped ZnO/cellulose composites based on tunable cellulose fiber sizes for enhancing photocatalytic properties’, *Carbohydrate Polymers*, vol. 250, p. 116873, Dec. 2020, doi: 10.1016/j.carbpol.2020.116873.

[7] M. Liu, Y. Wan, C. Zhu, G. Chen, and X. Li, ‘FeNi bimetallic modified pg-C3N4-x and its photoelectrocatalytic hydrogen production coupling anodic oxidation’, *Separation and Purification Technology*, vol. 357, p. 130142, May 2025, doi: 10.1016/j.seppur.2024.130142.

[8] S.-W. Zhao, M. Zheng, X.-H. Zou, Y. Guo, and Q.-J. Pan, ‘Self-Assembly of Hierarchically Structured Cellulose@ZnO Composite in Solid–Liquid Homogeneous Phase: Synthesis, DFT Calculations, and Enhanced Antibacterial Activities’, *ACS Sustainable Chem. Eng.*, vol. 5, no. 8, pp. 6585–6596, Aug. 2017, doi: 10.1021/acssuschemeng.7b00842.
